# Evaluation of Automatic Segmentation Model With Dosimetric Metrics for Radiotherapy of Esophageal Cancer

**DOI:** 10.3389/fonc.2020.564737

**Published:** 2020-09-29

**Authors:** Ji Zhu, Xinyuan Chen, Bining Yang, Nan Bi, Tao Zhang, Kuo Men, Jianrong Dai

**Affiliations:** National Cancer Center/National Clinical Research Center for Cancer/Cancer Hospital, Chinese Academy of Medical Sciences and Peking Union Medical College, Beijing, China

**Keywords:** automatic segmentation, dosimetric evaluation, esophageal cancer, deep learning, organs at risk, radiotherapy

## Abstract

**Background and Purpose:** Automatic segmentation model is proven to be efficient in delineation of organs at risk (OARs) in radiotherapy; its performance is usually evaluated with geometric differences between automatic and manual delineations. However, dosimetric differences attract more interests than geometric differences in the clinic. Therefore, this study aimed to evaluate the performance of automatic segmentation with dosimetric metrics for volumetric modulated arc therapy of esophageal cancer patients.

**Methods:** Nineteen esophageal cancer cases were included in this study. Clinicians manually delineated the target volumes and the OARs for each case. Another set of OARs was automatically generated using convolutional neural network models. The radiotherapy plans were optimized with the manually delineated targets and the automatically delineated OARs separately. Segmentation accuracy was evaluated by Dice similarity coefficient (DSC) and mean distance to agreement (MDA). Dosimetric metrics of manually and automatically delineated OARs were obtained and compared. The clinically acceptable dose difference and volume difference of OARs between manual and automatic delineations are supposed to be within 1 Gy and 1%, respectively.

**Results:** Average DSC values were greater than 0.92 except for the spinal cord (0.82), and average MDA values were <0.90 mm except for the heart (1.74 mm). Eleven of the 20 dosimetric metrics of the OARs were not significant (*P* > 0.05). Although there were significant differences (*P* < 0.05) for the spinal cord (D2%), left lung (V10, V20, V30, and mean dose), and bilateral lung (V10, V20, V30, and mean dose), their absolute differences were small and acceptable for the clinic. The maximum dosimetric metrics differences of OARs between manual and automatic delineations were ΔD2% = 0.35 Gy for the spinal cord and ΔV30 = 0.4% for the bilateral lung, which were within the clinical criteria in this study.

**Conclusion:** Dosimetric metrics were proposed to evaluate the automatic delineation in radiotherapy planning of esophageal cancer. Consequently, the automatic delineation could substitute the manual delineation for esophageal cancer radiotherapy planning based on the dosimetric evaluation in this study.

## Introduction

One of the challenges in radiotherapy is the accurate delineation of organs at risk (OARs). Various delineation techniques are used by different professionals. Automatic segmentation of OARs with artificial intelligence has great application value for treatment planning in radiotherapy because of its high efficiency and advanced delineation accuracy.

Several studies focused on the geometric evaluation between manual and automatic segmentation delineations. The geometric evaluation compares the similarity between different delineation methods by Dice similarity coefficient (DSC) and mean distance to agreement (MDA). The DSC evaluates the similarity of two delineations by comparing the overlap area. The MDA shows the average distance of outline points between the overlap volume of two delineations. Liang et al. (1) evaluated the quality of automatic delineation by using geometric discrepancies in head and neck OARs. Ahn et al. (2) demonstrated that the deep convolution neural network methods could provide an effective and efficient way to delineate OARs for liver cancer. For automatic delineation in the thorax, Yang et al. (3) reported that several institutions participated in the thoracic automatic segmentation challenge organized by the American Association of Physicists in Medicine in 2017. The DSC scores of the left lung, right lung, heart, and spinal cord were 0.956 ± 0.019, 0.955 ± 0.019, 0.931 ± 0.015, and 0.862 ± 0.038, respectively (3). Lustberg et al. (4) showed their geometric evaluation of automatic delineations for lung cancer in 2018: the spinal cord (median Dice score, 0.83), the lungs (median Dice score, >0.95), and the heart (median Dice score, >0.90). Dong et al. (5) addressed that the averaged DSC scores for the left lung, right lung, spinal cord, and heart were 0.97, 0.97, 0.90, and 0.87, correspondingly, in 2019. Therefore, thoracic OARs including the spinal cord, lungs, and heart could be segmented accurately by the automatic delineation method (5).

However, the primary concern in radiotherapy is not the delineation accuracy but the dosimetric impacts of the delineation. To show that a model successfully segments the OARs in geometry is not sufficient to confirm its reliability for radiotherapy utilization. Vinod et al. (6) believed that it is similar to geometric evaluation of different delineations; there was no standardized method of dosimetric comparison of delineations. Accordingly, a quantitative system to evaluate both the dosimetric and geometric parameters of manual and automatic delineation-generated plans becomes necessary. Fung et al. (7) showed their studies about geometric discrepancies and dose impact between manually and automatically delineated OARs in nasopharyngeal carcinoma in a creative manner. Especially, Fung et al. (7) evaluated manual and automatic delineations using dosimetric discrepancies, which include maximum dose, D1 cc, and D50%. However, their study did not evaluate the automatic delineation using clinical dosimetric metrics.

No study on the impact of dosimetric metrics between manual and automatic delineations has been conducted yet, specifically in esophageal cancer. Further, esophageal cancer is common around Asia, especially in eastern Asia. More than 700 esophageal cancer patients are treated by radiotherapy in our department every year. Therefore, automatic delineation of esophageal cancer will play an important role in the clinic. This study introduces a dosimetric evaluation method to substitute the geometric evaluations on automatic delineation for esophageal cancer VMAT radiotherapy.

## Methods

### Data Acquisition

The data consisted of 19 stage III/IV esophageal cancer patients who were treated from December 2018 to July 2019 in our department. The inclusion criteria of patients were proven and diagnosed histologically as esophageal cancer according to the guideline of the TNM staging system. The detailed demographics of the included patients are shown in Table 1. All patients were set up with the supine position on a commercial “bellyboard” and immobilized using a thermoplastic mask. The data of planning computed tomography (CT) images were acquired from the Somatom Definition AS 40 (Siemens Healthcare, Forchheim, Germany) or the Brilliance CT Big Bore (Philips Healthcare, Best, the Netherlands) systems on helical scan mode. CT images were reconstructed using a matrix size of 512 × 512 and a slice thickness of 5 mm. The delineation of OARs was delineated on CT images according to RTOG 0617 and RTOG 1106 standard contouring atlas (8, 9). Meanwhile, the delineation of OARs was delineated and approved by senior clinicians for this study.

**Table 1:** The Clinical data of patients.

**Characteristic**		***N* (*n* = 19)**
Age	Median	59.05 ± 8.26
Gender	Male	16
	Female	3
Pathology	Squamous cell carcinoma	18
	Small cell carcinomas	1
Primary location	Cervical	0
	Thoracic	19
Chemotherapy	Neoadjuvant	8
	Concurrent	8
	None	3
T stage	T3	11
	T4	8
N stage	N1	17
	N2	1
	N3	1
M stage	M0	14
	M1	5

*The values in the “Age” row represent as mean ± standard deviation*.

### Architecture of Segmentation Network

Five hundred patients diagnosed with thorax tumor who received radiotherapy between 2011 and 2019 were enrolled for training the OAR-segmentation models. The OARs for segmentation included bilateral lungs, heart, spinal cord, and bilateral kidneys. Fifty patients from 2018 to 2019 were chosen randomly to validate the deep learning model. The validation set was used to assess the performance of the deep learning model.

**Figure 1:**
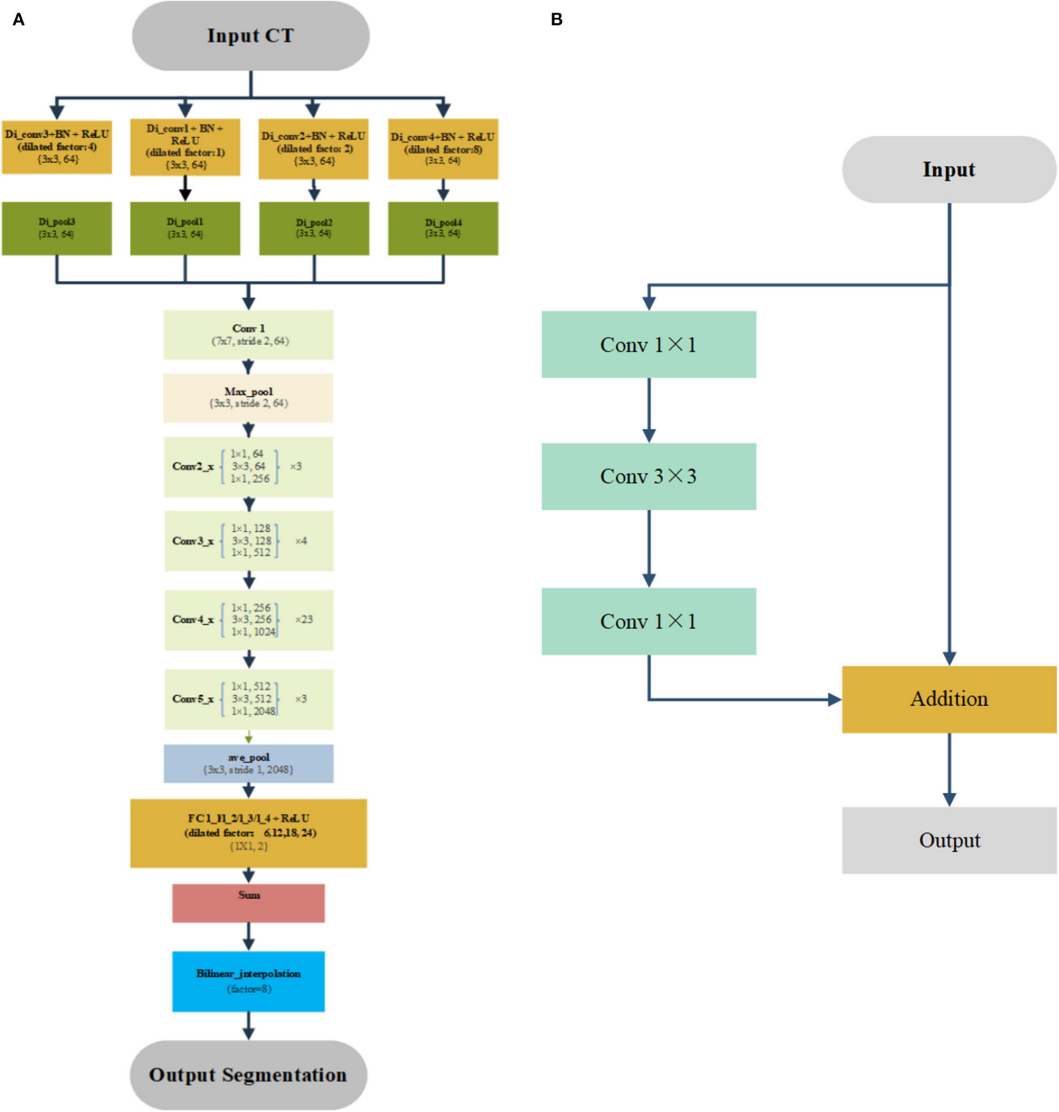
Architecture of automatic segmentation network **(A)** and example of the residual block **(B)**.

We used this previously published deep learning algorithm to segment the OARs for treatment planning (10). Figure 1 shows the detailed architectures. A four-stream dilated convolutional module was applied before the ResNet-101 networks. The advantage is that it can extract multiscale features from the original CT image with different dilated factors. The multiscale feature maps were added and feed forward to the ResNet-101, which has 101 weighted layers. Its characteristic is the use of several residual blocks to avoid gradients vanishing. An example of the residual block is shown in Figure 1B. It took a standard feed-forward convolutional network and added skipped connections that bypassed a few convolutional layers at a time. Each bypass gave rise to a residual block in which the convolutional layers predicted a residual that was added to the input tensor of the block. There were 3, 4, 23, and 4 such blocks in conv2, conv3, conv4, and conv5, respectively. The size of image was reduced to 1/8 of the original network with the down-sampling. Therefore, a bilinear interpolation was applied to the sum layer to recover the feature map to the original size for pixel-level classification.

### Experiments

The clinicians manually delineated the planning target volume, the planning gross tumor volume, and the OARs, including the spinal cord, spinal cord planning OAR volume (PRV), heart, left lung, right lung, and whole lung, as the ground truth (GT) set. The previously published deep learning model was used for this automatic segmentation task (10). The automatically delineated OARs included the spinal cord, spinal cord PRV, heart, left lung, right lung, and bilateral lung.

**Figure 2:**
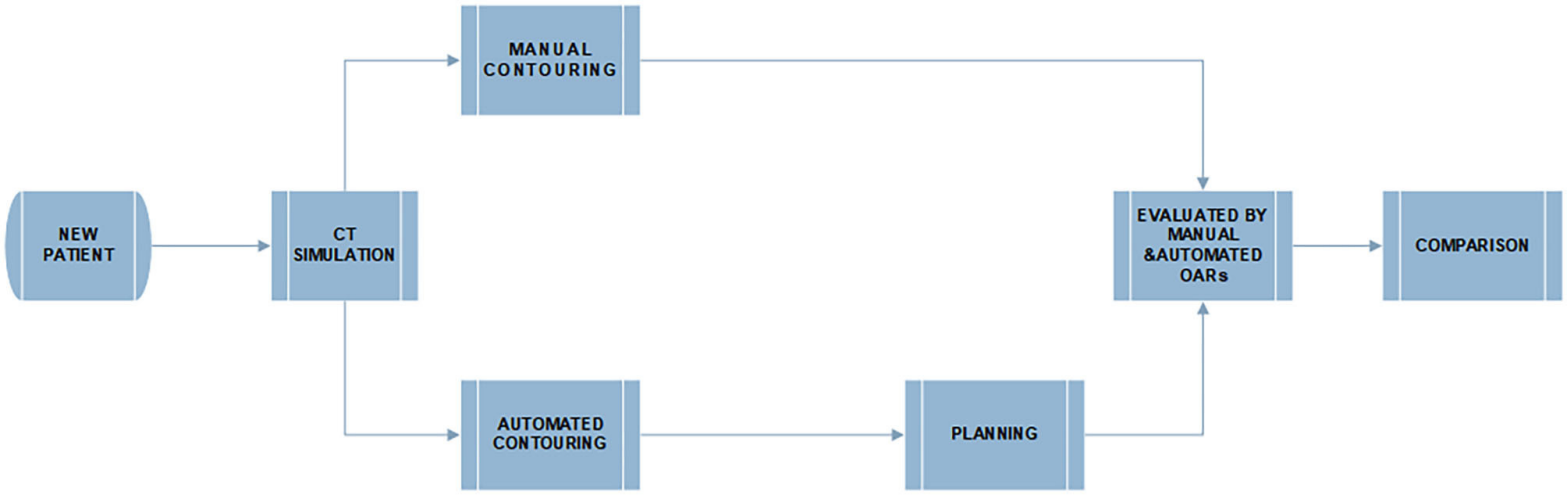
The flowchart of manual and deep learning automatic delineation-based plan evaluation experiment.

The work flowchart of this study is illustrated in Figure 2. The radiotherapy plans were designed and optimized with the manually delineated targets and the automatically delineated OARs. The dose constraints are followed by published guideline: the maximum dose of the spinal cord was ≤40 Gy, the spinal cord PRV was ≤45 Gy, V20 Gy of the bilateral lung was ≤25 or 30% in special cases, and the heart V30 and V40 Gy was ≤40 and ≤30%, respectively (8, 11). D (*x*%) means of the dose received by *x*% of the OARs volume. Dmean is defined as the average dose of OARs receiving. The V*x* Gy is defined as the volume of normal OARs receiving more than *x* Gy dose (10). Further, the clinically acceptable dose difference and volume difference of OARs between manual and automatic delineations should be <1 Gy and 1%, respectively. All the plans were evaluated and approved by senior clinicians.

Next, the dosimetric metrics of the plans were calculated and evaluated using the manual and automatic segmentation delineated OARs, separately. Finally, both manual and automatic delineations were compared with metrics of the geometry and clinical dosimetry.

### Evaluation

#### Geometric Metrics

The DSC and MDA were used in this study (7, 12, 13).

As shown above, the DSC is one of the geometric evaluation methods in this study, otherwise known as Sørensen–Dice coefficient (14), which is used to evaluate the similarity of two samples such as imaging and radiotherapy target volume segmentation.

$$\begin{array}{l} \text{DSC}\left(\text{A},\text{B}\right)=\frac{2|\text{A}\cap \text{B}|}{\left|\text{A}|+|\text{B}\right|} \end{array}$$

The DSC had values between 0 and 1 (0 = no overlap, 1 = complete overlap). *A* is the investigator (automatic) delineation, and *B* is the GT (manual) delineation.

The MDA indicates the average distance of outline points of the automatic contouring volume to the outline of reference manual delineation perfect overlap volume (15). The lower the values (mm) of MDA, the higher the correspondence between the automatic and manual contouring volumes (15).

#### Dosimetric Metrics

Radiotherapy plans were designed by using the Pinnacle^3®^ Radiation Therapy Planning software (version 9.1; Philips Medical Systems Inc., Fitchburg, MA, USA). The dosimetric parameters, including D2%, Dmean, V40, V30, V20, and V10 Gy, were used to evaluate the plan quality and OARs sparing.

The continuous variables were presented as the mean ± SD and should be rounded up to two decimal places, which are dependent on the normality of the data. Correspondingly, the paired *t*-test was used to compare the variables between the manually and automatically delineated methods. All of the statistical analyses were conducted using the IBM SPSS Statistics software (version 25.0; IBM Inc., Armonk, NY, USA). All paired *t*-tests were two-sided. The difference between manually (GT) and automatically delineated dosimetric metrics was considered as statistically significant when the paired *t-*test showed *P* < 0.05.

The dosimetric characteristics of OARs were gauged by the conformity index (CI) and homogeneity index (HI) (11, 16, 17). CI of target volume is defined as following equation (11):

$$\begin{array}{l} \text{CI}=\frac{{\text{TVPTV}}^{2}}{\text{TV}*\text{PTV}} \end{array}$$

where TV is the volume of prescribed isodose line enclosed volume. PTV is the volume of targets. TVPTV represents the overlap volume between volume of targets volume and the prescribed isodose line enclosed volume.

HI of target volume is a simple scoring tool that quantifies dose homogeneity in the target volume. It is therefore used to evaluate and compare the dose distributions of various treatment plans (11, 17).

The formula of HI is suggested by the ICRU 83 report as the following equation:

$$\begin{array}{l} \text{HI}=\frac{\text{D}2\%-\text{D}98\%}{\text{D}50\%} \end{array}$$

The D2%, D98%, and D50% are doses delivered to 2, 98, and 50% volume of target volume, respectively. The closer the HI value approaches 0, the better homogeneity of target volume is (11).

## Results

### Geometric Metrics

The performance of our deep learning model is shown in Table 2. The MDAs of the left lung, right lung, bilateral lung, heart, spinal cord, spinal cord PRV, left kidney, and right kidney were 0.68 ± 0.13, 0.82 ± 0.20, 0.73 ± 0.12, 1.87 ± 0.69, 0.79 ± 0.15, 0.80 ± 0.15, 1.12 ± 0.31, and 1.01 ± 0.29 mm, respectively. The segmentation accuracy values for the left lung, right lung, bilateral lung, heart, spinal cord, spinal cord PRV, left kidney, and right kidney in validation set are shown as follows: DSC: 0.97 ± 0.01, 0.97 ± 0.01, 0.97 ± 0.01, 0.93 ± 0.03, 0.83 ± 0.03, 0.91 ± 0.02, 0.93 ± 0.02, and 0.93 ± 0.02, respectively. It implied that the deep learning model was reliable in automatically delineated OARs for esophageal cancer.

**Table 2:** The geometric characteristic of OARs of validation set.

**OARs**	**MDA (mm)**	**DSC**
Lung L	0.68 ± 0.13	0.97 ± 0.01
Lung R	0.82 ± 0.20	0.97 ± 0.01
Lung all	0.73 ± 0.12	0.97 ± 0.01
Heart	1.87 ± 0.69	0.93 ± 0.03
Spinal cord	0.79 ± 0.15	0.83 ± 0.03
Spinal cord PRV	0.80 ± 0.15	0.91 ± 0.02
Kidney L	1.12 ± 0.31	0.93 ± 0.02
Kidney R	1.01 ± 0.29	0.93 ± 0.02

*MDA, mean distance to agreement; DSC, Dice similarity coefficient*.

Table 3 shows the mean value and standard deviation of the DSC and MDA, respectively. It also shows that the MDA of the spinal cord and spinal cord PRV was shorter than that of the left lung, right lung, bilateral lung, and heart. The MDAs of the left lung, right lung, bilateral lung, heart, spinal cord, and spinal cord PRV were 0.82 ± 0.21, 0.90 ± 0.31, 0.85 ± 0.20, 1.74 ± 0.85, 0.75 ± 0.22, and 0.74 ± 0.22 mm, respectively.

**Table 3:** The geometric characteristic of OARs between manual and deep learning automatic delineation-based plan.

**OARs**	**MDA (mm)**	**DSC**
Lung L	0.82 ± 0.21	0.97 ± 0.01
Lung R	0.90 ± 0.31	0.97 ± 0.01
Lung all	0.85 ± 0.20	0.97 ± 0.01
Heart	1.74 ± 0.85	0.93 ± 0.04
Spinal cord	0.75 ± 0.22	0.84 ± 0.04
Spinal cord PRV	0.74 ± 0.22	0.92 ± 0.02

*MDA, mean distance to agreement; DSC, Dice similarity coefficient*.

**Figure 3:**
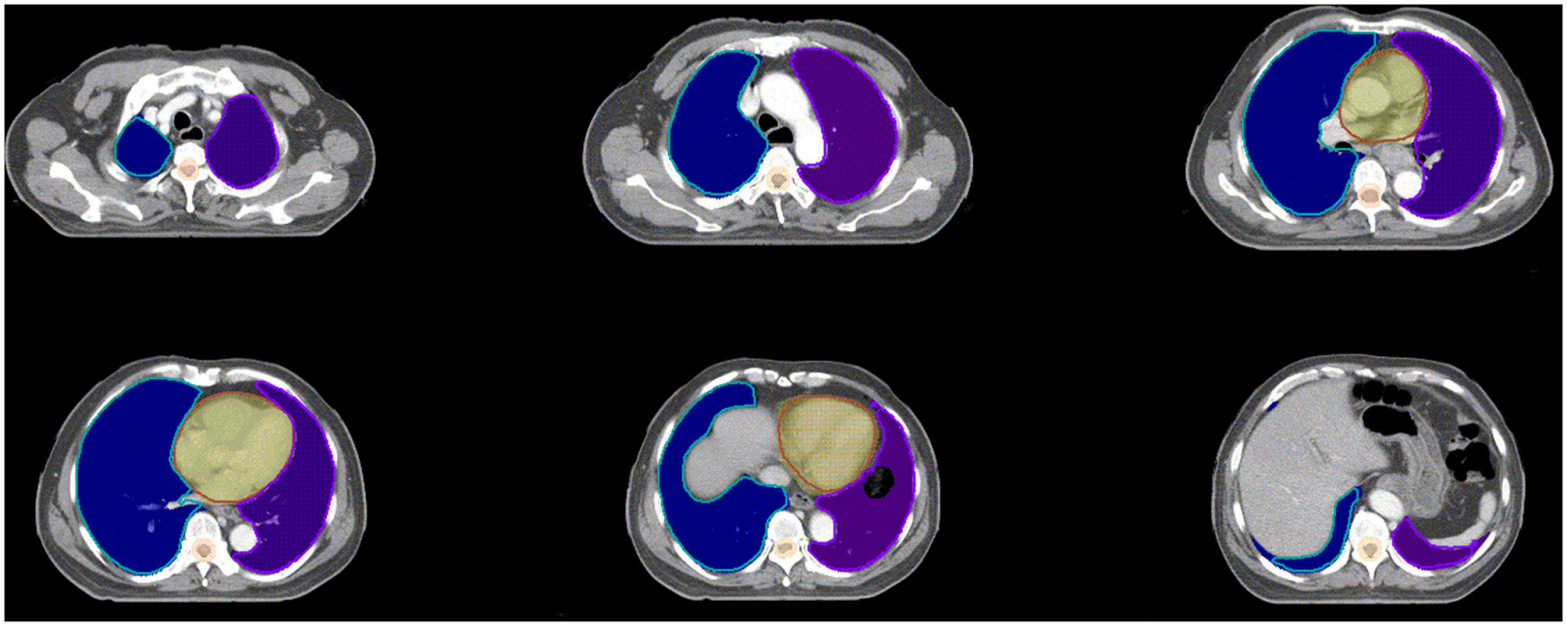
Examples of the segmentation. Color wash: manual segmentation; Line: automatic segmentation.

The spinal cord DSC value was 0.84 ± 0.04, which was the lowest value in all of six OARs. The OARs including the left lung, right lung, bilateral lung, and heart showed good performance in DSC evaluation. The segmentation accuracy values for the spinal cord PRV, heart, left lung, right lung, and bilateral lung (lung all) are shown as follows: DSC: 0.92 ± 0.02, 0.93 ± 0.04, 0.97 ± 0.01, 0.97 ± 0.01, and 0.97 ± 0.01, correspondingly. Examples of the segmentation results are shown in Figure 3, which illustrates that the segmentation was in good agreement with the manual delineation.

### Dosimetric Metrics

Table 4 shows the paired *t*-test confidence interval of the spinal cord and spinal cord PRV D2% conversely. The dose difference of spinal cord D2% between manual and automatic delineations was significant. The V30, V40, and mean dose of the heart were insignificant. All of the corresponding paired *t*-test confidence interval data of the right lung presented were insignificant. By contrast, the *P*-value of the left lung was <0.001, except for the V5 of the left lung. For the bilateral lung, the corresponding V30, V20, V10, and mean of manual delineation were significantly higher than those of the automatic delineation. V5 of the bilateral lung was insignificant, with interval confidence of 0.44. Except the V30, V40, and mean of heart, as well as the V5 of right and left lung, most of the dosimetric metrics of manual delineation OARs were found to be relatively significantly higher than automatic delineation OARs. For all of patients' OARs, including spinal cord and lungs, both the manual and automatic delineation plans were able to meet the clinical dose constraints. Only the heart V30 of two patients (#1: manual: 40.71%, automatic: 41.09%; #2: manual: 49.56%, automatic: 48.02%) could not meet the clinical dose constraints because of their targets close to their heart. However, the heart V30 of these two patients was still variation-acceptable in the clinic.

**Table 4:** The paired *t*-test outcome of the dosimetric characteristic of OARs between manual and deep learning automatic delineation-based plan.

**Dosimetric metrics**		**GT**	**AI**	***P*-value**
Spinal cord	D2% (Gy)	36.08 ± 0.41	35.73 ± 0.41	<0.01
Spinal cord PRV	D2% (Gy)	40.25 ± 0.43	40.42 ± 0.30	0.55
Heart	V30 (%)	28.60 ± 4.06	28.70 ± 4.09	0.87
	V40 (%)	14.68 ± 2.19	15.00 ± 2.28	0.48
	Mean (Gy)	20.54 ± 2.71	20.64 ± 2.76	0.65
Lung all	V30 (%)	8.63 ± 2.69	8.23 ± 2.73	0.02
	V20 (%)	15.81 ± 4.95	15.63 ± 4.99	<0.01
	V10 (%)	26.47 ± 8.27	26.28 ± 8.28	0.04
	V5 (%)	41.05 ± 12.76	41.48 ± 13.18	0.44
	Mean (Gy)	9.26 ± 2.55	9.21 ± 2.57	0.04
Lung L	V30 (%)	10.24 ± 4.86	10.01 ± 4.95	<0.01
	V20 (%)	18.55 ± 8.28	18.28 ± 8.38	<0.01
	V10 (%)	30.53 ± 12.02	30.31 ± 12.07	0.04
	V5 (%)	45.84 ± 16.26	45.88 ± 16.45	0.73
	Mean (Gy)	10.31 ± 3.68	10.21 ± 3.71	<0.01
Lung R	V30 (%)	7.31 ± 3.75	7.32 ± 3.79	0.90
	V20 (%)	13.55 ± 5.35	13.45 ± 5.28	0.41
	V10 (%)	23.11 ± 7.83	22.97 ± 1.76	0.42
	V5 (%)	37.04 ± 11.71	37.01 ± 11.74	0.89
	Mean (Gy)	8.40 ± 2.51	8.38 ± 2.51	0.75

**Figure 4:**
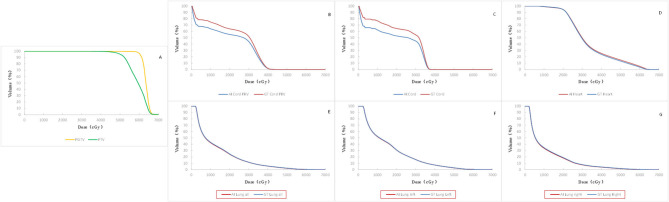
Comparison of mean DVH of all the 19 cases. Red solid line: automatic segmentation Blue solid line: manual segmentation including DVH of targets **(A)**, spinal cord PRV **(B)**, spinal cord **(C)**, heart **(D)**, bilateral lung **(E)**, left lung **(F)**, and right lung **(G)**.

The mean dose volume histogram curves (Figure 4) of plans with manual and automatic segmentation were close for most of the OARs. Table 4 shows that the maximum dosimetric metrics differences of OARs between manual and automatic delineations were ΔD2% = 0.35 Gy for spinal cord and ΔV30 = 0.4% for bilateral lung, which corresponded with the clinical criteria in this study. The CIs of PTV and PGTV were 0.73 ± 0.083 and 0.83 ± 0.071, respectively. In addition, The HIs of PTV and PGTV were 0.27 ± 0.020 and 0.085 ± 0.014, correspondingly.

## Discussion

The geometric results illustrate that the segmentation was in close agreement with the manual delineation when considering the DSC. Because of the lack of relevant reports on automatic delineation in esophageal cancer, we can compare them with thoracic OAR automatic delineation reports. Yang et al. (3) addressed the mean value of DSC in thoracic automatic segmentation. Their data included the left lung, right lung, heart, and spinal cord whose DSC values were 0.956 ± 0.019, 0.955 ± 0.019, 0.931 ± 0.015, and 0.862 ± 0.038, respectively. Dong et al. (5) addressed that the averaged DSC scores for the left lung, right lung, spinal cord, and heart OARs were 0.97, 0.97, 0.90, and 0.87, respectively, in 2019. The lowest DSC value in all of the six OARs is the spinal cord (0.84 ± 0.04) in our study. The OARs including the spinal cord PRV (0.92 ± 0.02), left lung (0.97 ± 0.01), right lung (0.97 ± 0.01), lung all (0.97 ± 0.01), and heart (0.93 ± 0.04) showed good performance in DSC evaluation. Therefore, OARs including the spinal cord, lungs, and heart were accurately segmented by the automatically delineated method in this study.

The finding of CIs and HIs above indicate that each radiotherapy plan has good conformity and homogeneity, which can fully meet the clinical requirement. The dosimetric metrics of the spinal cord PRV, heart, and right lung between manual and automatic delineations show no difference in this study. The corresponding *P*-value of the dosimetric metrics between the manual and automatic delineation sets shows an insignificant value that could indicate their equivalent nature. The automatic delineation of the heart and right lung shows a relative equivalent quality in dosimetric metrics when compared with manual delineation.

**Figure 5:**
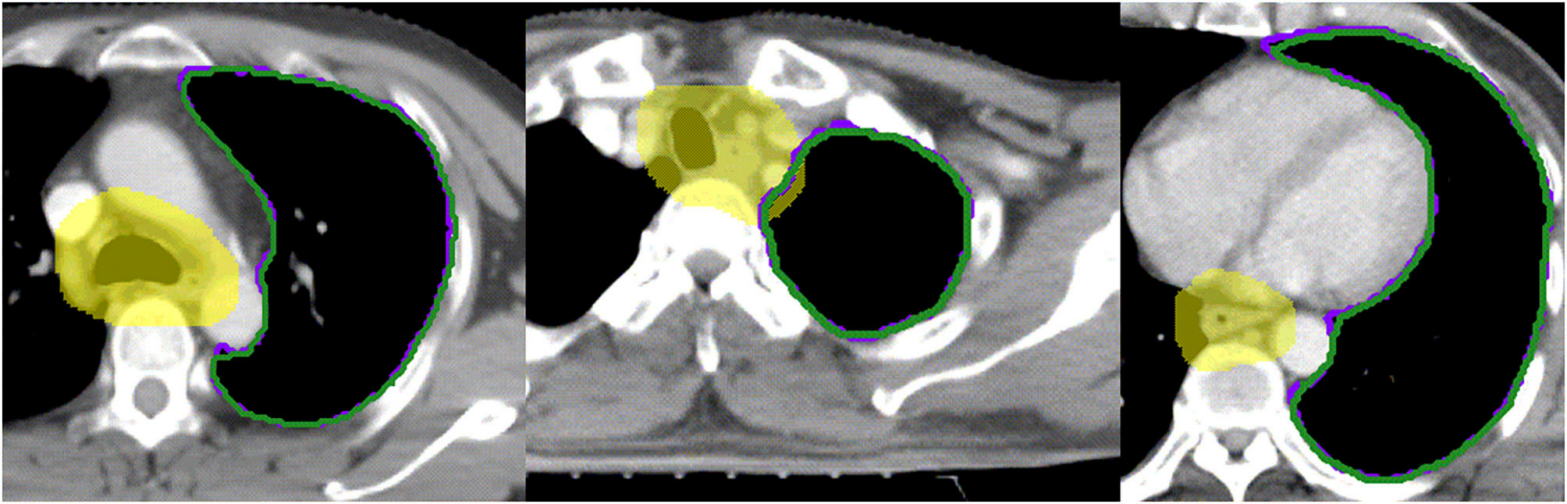
Examples of the segmentation of left hilum. Yellow color wash: PTV; Purple line: manual segmentation; Green line: automatic segmentation.

By contrast, the D2% of the spinal cord and the mean dose, V10, V20, and V30 of the left and bilateral lungs show significant value (*P* < 0.05). Based on our knowledge, the hilum of lung is a steep dose falloff area in esophageal cancer radiotherapy. As an observation of manually and automatically delineated OARs in Figure 5, the automatically delineated left lung shows a distortion around the left hilum comparing with manual delineation. Therefore, dosimetric metrics of the left and bilateral lungs show significant values in paired *t*-test. The difference of V5 was insignificant for right, left, and bilateral lung. Considering the toxicity of radiotherapy in the lung, Luna et al. (18) reported that the lung V5 (>43.6%) could predict the presence of radiation pneumonitis consistently. The mean V5 values of manually and automatically delineated bilateral lung were <43.6%, which implies a lower risk of severe radiation pneumonitis in this study. By the review of studies and radiotherapy guideline, the dose impact is notable in the steep dose fall area (7). The 2% volume of the spinal cord (manual 1.13 ± 0.35 cc vs. automatic 1.18 ± 0.26 cc) was relatively equal to a voxel of CT in the planning system (1 cm × 1 cm × 1 cm). The average 2% volumes of the spinal cord PRV were 4.08 ± 0.75 and 4.25 ± 0.66 cc for manual and automatic delineations, respectively. D2% of the spinal cord was relatively equal to the point dose in radiotherapy planning. Therefore, the D2% of the spinal cord shows significant value (*P* < 0.05). The dosimetric metrics of spinal cord PRV is more important in this study because the PRV is recommended for the structures of the nervous system including the spinal cord (11, 19).

Although the dose differences of the spinal cord, left lung, and bilateral lung are significant, their absolute difference is small and acceptable for clinical use. Table 4 shows that the maximum dosimetric metrics differences of OARs between manual and automatic delineations are <1 Gy (spinal cord, ΔD2% = 0.35 Gy) and 1% (bilateral lung, ΔV30 = 0.4%). The dose difference and volume difference of OARs had no impact on the radiation toxicity of each OARs, because the OARs of both manual and automatic delineations do not approach their maximum tolerance in this study (8). Chicas-Sett et al. (20) reported that the manual delineation also depends on intraobserver or interobserver deviations, which leads to dosimetric difference and organ-sparing failure.

As shown in the results, the dosimetric metrics of manual delineation OARs were found to be relatively significantly higher or lower than automatic delineation OARs. However, the dosimetric metrics of manually delineated OARs for each patient did not show a directional higher or lower trend than automatic delineation OARs. This result implies that the dosimetric metrics of manual and automatic delineation methods conform to Gaussian distribution, which had been proved in paired *t*-test.

Although the deep learning segmentation model shows outperformance, there are still limitations to this study. In order to improve the performance of automatic delineation model, larger training data are recommended in future work. Further, three-dimensional radiography information will be valuable in the architecture of deep learning model. As shown in Figure 5, the automatically delineated left lung illustrates a distortion around the left hilum. This limitation might be overcome with the combination of threshold method and automatic delineation.

## Conclusion

The findings of this study showed that the geometric evaluation between manual and automatic delineations was not enough in clinical applications. Dosimetric metrics were proposed to assess the automatic delineation in radiotherapy planning of esophageal cancer. Based on the dosimetric evaluation in this study, the manual delineation for esophageal cancer radiotherapy can be substituted by automatic delineation.

## Data Availability Statement

The datasets presented in this article are not readily available because of privacy requirements of the hospital. Requests to access the datasets should be directed to the corresponding author.

## Ethics Statement

This study was carried out in accordance with the Declaration of Helsinki and was approved with exemption from informed consent by the Independent Ethics Committee of Cancer Hospital, Chinese Academy of Medical Sciences.

## Author Contributions

All authors discussed and conceived of the study design. JZ and XC trained the deep learning models, performed data analysis, and drafted the manuscript. BY, NB, and TZ helped to collect the data and evaluate radiotherapy planning. JD and KM guided the study and participated in discussions and preparation of the manuscript. All authors read, discussed, and approved the final manuscript.

## Conflict of Interest

The authors declare that the research was conducted in the absence of any commercial or financial relationships that could be construed as a potential conflict of interest.

## Abbreviations

OARs: Organs at risk
VMAT: Volumetric modulated arc therapy
CNN: Convolutional neural network
DSC: Dice similarity coefficient
MDA: Mean distance to agreement
DCNN: Deep convolution neural network
CT: Computed tomography
PTV: Planning target volume
PGTV: Planning gross tumor volume
PRV: Planning organ at risk volume
DVH: Dose volume histogram.
